# Biotic Stress Shifted Structure and Abundance of *Enterobacteriaceae* in the Lettuce Microbiome

**DOI:** 10.1371/journal.pone.0118068

**Published:** 2015-02-25

**Authors:** Armin Erlacher, Massimiliano Cardinale, Martin Grube, Gabriele Berg

**Affiliations:** 1 Institute of Environmental Biotechnology, Graz University of Technology, Petersgasse 12, 8010, Graz, Austria; 2 Institute of Plant Sciences, University of Graz, Holteigasse 6, 8010, Graz, Austria; University of Osnabrueck, GERMANY

## Abstract

Lettuce cultivars are not only amongst the most popular vegetables eaten raw, they are also involved in severe pathogen outbreaks world-wide. While outbreaks caused by *Enterobacteriaceae* species are well-studied, less is known about their occurrence in natural environments as well as the impact of biotic stress. Here, we studied the ecology of the human health-relevant bacterial family *Enterobacteriaceae* and assessed the impact of biotic disturbances by a soil-borne phytopathogenic fungus and *Gastropoda* on their structure and abundance in mesocosm and pot experiments. Using a polyphasic approach including network analyses of 16S rRNA gene amplicon libraries, quantitative PCR and complementary fluorescence *in situ* hybridization (FISH) microscopy we found substantial yet divergent *Enterobacteriaceae* communities. A similar spectrum of 14 genera was identified from rhizo- and phyllospheres but the abundance of *Enterobacteriaceae* was on average 3fold higher in phyllosphere samples. Both stress factors shifted the bacterial community of the leaf habitat, characterized by increases of species abundance and diversity. For the rhizosphere, we observed significant structural shifts of *Enterobacteriaceae* communities but also a high degree of resilience. These results could be confirmed by FISH microscopy but it was difficult to visualize phyllosphere communities. Additional inoculation experiments with *Escherichia coli* as model revealed their presence below the wax layer as well as in the endosphere of leaves. The observed presence influenced by stress factors and the endophytic life style of *Enterobacteriaceae* on lettuce can be an important aspect in relation to human health.

## Introduction

Lettuce (*Lactuca sativa* L.) is regarded highly re-esteemed as an important staple of a balanced, healthy diet. Globally, lettuce consumption has increased during the past several years [1], and in 2012 lettuce and chicory production was noticed with 24.9 Mio t (FAOSTAT). Pathogen outbreaks associated with lettuce, caused mainly by members of *Enterobacteriaceae*, are a major problem for human health world-wide; therefore they are well-studied [1–5]. In the past, *Enterobacteriaceae* are known as inhabitants of the intestines of animals and humans with *Escherichia coli* as long-studied model bacterium, pathogens, such as *E*. *coli* pathovars, *Yersinia pestis*, *Klebsiella*, *Salmonella* and *Shigella* spp. but also symbionts, suggesting that they are important for human health [4]. Recently, it was reported that *Enterobacteriaceae* are also indigenous members of several plant microbiomes [2,4]. However, less is known about the natural occurrence and ecology of this health-relevant bacterial group on lettuce. As with all plants, lettuce provides habitats for specific microbes as shown by Rastogi et al. [6,7]. High bacterial abundances of 10^5^–10^6^ CFU g^-1^ fw and diversities with a substantial proportion of *Enterobacteriaceae* were identified in the phyllosphere of pot-grown lettuce [6–8]. Despite these studies, less is known about the variability and stability of the lettuce microbiome under various conditions.

Plants are colonized by specific microbiomes, which contribute to the growth, health, and fitness of their hosts [9,10]. This is due to specific conditions and exudates, as well as defensive mechanisms, and all these factors trigger species- and even cultivar-specific microbiomes [11,12]. Moreover, each individual plant harbors different microbial communities specific to the organs of each individual plant, mainly the phyllosphere (leaves) and rhizosphere (roots) [10]. Although the majority of microorganisms may be characterized as having a positive interaction with their hosts, human and plant pathogens are also part of the plant-associated microbiota [13–15]. Bacteria are key players in plant-associated ecosystems, and therefore it is crucial to identify the structure and function of bacterial communities. It is additionally important to understand the effect of biotic disturbances, which often occur in nature as well as under agricultural conditions, and their consequential structural and functional responses [16]. The basidiomycetous fungus *Rhizoctonia solani* Kühn cannot only cause severe diseases on lettuce with yield losses up to 70%, it can also colonize lettuce plants without or with only slight symptoms [17]. Slugs and snails (*Gastropoda*) are widespread agricultural pests and are also important for the cultivation of lettuce of both commercially and privately grown lettuce [18]. Over the past decade, the application of novel technologies has not only revolutionized our knowledge of the microbiome; it has drastically changed our view on pathogens and diseases [15,19]. For more than a century, pathogens have been defined as causative agents of diseases in accordance with Koch’s postulates, which were later added by molecular criteria [19]. In terms of the human microbiome, however, we have learned that pathogen outbreaks are associated with shifts of the whole community including shifts of supporting pathogens [20–22]. In contrast to Chowdhury et al. [23] who applied the 16S rRNA gene-based fingerprinting method terminal restriction fragment length polymorphism (T-RFLP), we were able to confirm the finding for the plant microbiome by deep sequencing of the microbiota. Both the phytopathogenic fungus *R*. *solani* and its antagonistic counterpart *Bacillus amyloliquefacies* FZB42 shifted the bacterial community in lettuce [24]. Moreover, we found indications that especially the enterobacterial fraction was highly affected by the pathogen [24]. Therefore, we developed the hypothesis that biotic stress induces shifts in the lettuce-associated *Enterobacteriaceae* community.

Our objective was to study *Enterobacteriaceae* communities on lettuce plants and to assess the impact of biotic stresses on them. We evaluated the influence of the pathogenic fungus *Rhizoctonia solani* and *Gastropoda* on the structure and the abundance of *Enterobacteriaceae* in the rhizo- and phyllosphere of lettuce (*Lactuca sativa* L. var. *capitata*) in mesocosm and pot experiments using a combined approach including network analyses of 16S rRNA gene amplicon libraries, quantitative PCR and FISH microscopy.

## Materials and Methods

### Experimental design and sampling

We investigated the impact of *R*. *solani* AG1-IB on the lettuce-associated microbiota in the first experiment (E1). The phytopathogenic fungus *R*. *solani* was applied to young seedlings of *L*. *sativa* var. *capitata* in randomized block systems and was grown further under growth chamber conditions (20/15°C, light 16/8 h (day/night). Lettuce was planted at two-leaf stage into pots (500 ml) filled with the same substrate sand mixture used for the seedling trays at a 1:1 ratio (v/v) (Fruhstorfer Einheitserde Typ P, Vechta, Germany; chemical analysis (mg l^-1^): N = 120, P = 120, K = 170, Mg = 120, S = 100, KCl = 1, organic substance = 167, peat = 309, pH 5.9). The pots were watered daily and fertilized (0.2% Wuxal TOP N, Wilhelm Haug GmbH and Co. KG, Düsseldorf, Germany) weekly. The sampling was carried out four weeks after inoculation with *R*. *solani*. In the treatments with pathogen inoculation, the substrate mixture was inoculated with ten *R*. *solani*-infested barley kernels and incubated at 25°C for one week until planting of lettuce into the pots. The microenvironments phyllosphere (leaves of each plant) and rhizosphere (roots of each plant including adhering soil) were separated. Six independent replicates were investigated for each microhabitat as well as for healthy plants (C_P/R) and plants showing severe bottom rot symptoms caused by *R*. *solani* indicated by brown lesions on petioles and the outer leaves (RS_P/R). The pot experiments were done at the Leibniz Institute of Vegetable and Ornamental Crops (Grossbeeren, Germany).

In the second experiment (E2), we developed an approach to study the impact of *Gastropoda* exposition on the lettuce (*L*. *sativa* var. *capitata*) microbiome in steady state glass tank mesocosms. The whole experimental procedure was conducted following customized methods to ensure no microbial carryover during transplantation, sample harvest or watering. Two glass tanks (50x30x30 cm) with a constitutional volume of 45 l were filled to one quarter capacity with identical homogenized organic potting soil (Dehner Bio Aussaat- und Kräutererde, Rain Germany; chemical analysis (mg l^-1^): N = 150, P = 180, K = 750, Mg = 150, S = 220, organic substance = 85%, pH 5.8). Twenty-two seedlings (*Lactuca sativa* var. *capitata*) were purchased at a local organic market, four plants were further separated (phyllosphere/rhizosphere) and processed for DNA extraction (C_T0), the remaining 18 plantlets were equally deployed in the two mesocosms. Lastly, the tanks were covered using a metal frame, coated with a mosquito mesh. The second sampling (C_T1) of four replicates (two plants/mesocosm) was conducted 13 days post planting. The sampling was carried out to determine effects and adaptions of the microbiota within the lettuce growth and establishment phase. The third sampling (C/G_T2) was carried out in the same way to the second sampling, five days later. Between the sampling C_T1 and C/G_T2 we enclosed 12 gastropods, members of *Limacidae* and *Arionidae* and snails (*Cepaea* and *Helicidae*) from the local environment to one mesocosm (G). The last sampling (C/G_T3) was performed six weeks after C/G_T2, to investigate sustainable changes in the microbial structure (gastropods were removed after G_T2).

From each experiment, four independent replicates consisting of 15–20 leaves or roots were collected and stored separately, placed into sterile plastic bags, and transported to the laboratory.

### Metagenomic DNA extraction

The microbial fraction associated with lettuce plants was extracted separately for the rhizo- and phyllospere [25]. From the plant material, 5 g of randomly cut tissues were physically disrupted with a sterile pestle and mortar and resuspended in 10 ml of 0.85% NaCl, and 2 ml of suspension were subsequently centrifuged (16,500 g, 20 min, 4°C). The obtained pellet was used for isolation of the total community DNA with the FastDNA SPIN Kit for Soil (MP Biomedicals, Solon, OH, USA). For mechanical lysis, the cells were homogenized twice in a FastPrep FP120 Instrument (Qbiogene, BIO101, Carlsbad, CA, USA) for 30s at a speed of 5.0 m sec^-1^ and treated according to the manufacturer’s protocol. Two technical DNA replicates from sampling were pooled for further processing.

### Microbial fingerprints by SSCP analysis of the 16S rRNA genes (PCR-SSCP)

In order to gain a first insight into structural changes and occurrence patterns of plants exposed to gastropods, fingerprinting by Single Strand Conformation Polymorphism (SSCP) [26] according to Rossman et al. [27] was performed. Briefly, bacterial 16S rRNA gene sequences were amplified by PCR using the universal bacterial primer pair Unibac-II-515f and Unibac-II-927rP [28] and for *Gammaproteobacteria* γ-prot 395f and γ-prot 871r [29]. After lambda exonuclease digestion the single stranded amplicons were separated using the TGGE Maxi System (Biometra, Göttingen, Germany) and visualized by silver staining. Evaluation of bacterial community profiles obtained by SSCP was performed by using the GelCompar program version 4.1 (Applied Maths, Kortrijk, Belgium).

### 454 pyrosequencing of 16S rRNA gene amplicons

The 16S rRNA gene fragments of all *Gammaproteobacteria* (E1) and *Enterobacteriaceae* (E2) were each amplified in a dual phase nested PCR approach using Multiplex Identifier (MID) tagged primers. Gammaproteobacterial 16S rRNA gene fragments were amplified using the V3–V5 primers γ-prot 395f and γ-prot 871rP [29] ensued by primer 515f and MID-modified 871r_454 [24,27]. For the PCR to investigate the enterobacterial diversity, the specific primers Entero-F234 and Entero-R1423 (V3–V8) for *Enterobacteriaceae* according to Binh et al. [30] were used. The nested PCR was carried out as described by Heuer et al. [31] using the primer pair F984 and R1378 modified for multiplex 454 sequencing according to the specification. The products of two independent PCR reactions from two independent samples were pooled and purified using the Wizard SV Gel and PCR Clean-Up System (Promega, Madison, WI, USA). Purified PCR products (200 ng each) were sequenced on a GS FLX 454 Titanium platform (Macrogen Korea, Seoul, South Korea).

Sequences were analyzed with the QIIME software version 1.7.0 [32]. MID, primer, and adapter sequences were removed, and the sequences were quality (minimal score: 50) and length filtered (minimal raw fragment length: *Gammaproteobacteria* ≥ 350; *Enterobacteriaceae* ≥ 430), followed by a denoising step (denoise wrapper and QIIME denoiser script). Chimeras and the remaining sequences of non-target, plastidal, and mitochondrial origins were removed. OTU (operational taxonomic unit) tables were created with UCLUST at a 97% cut-off level for the amplified *Gammaproteobacteria* and 98% cut-off level for the amplified enterobacteria, respectively. OTU tables were retrained on the latest green genes release (v13.5) and the *Gammaproteobacteria* OTU table (E1) was subsequently filtered to family level of *Enterobacteriaceae*. The datasets were rarified to the appropriate number of least reads per sample within each experiment to compute alpha and beta diversity indices. Unique reads were classified with the RDP classifier (v2.5) and normalized data were then produced from the relative abundance of taxa present in each sample based on a naїve Bayesian classifier [33]. Output sequences were classified as domain, phylum, family, and genus depending on the depth of reliable classifier assignments. Cytoscape (v2.8) was used to create an integrated model network [34] Statistical tests based on the OTU table were performed with the non-parametric ANOVA Kruskal Wallis test. This test is functionally an expansion of ANOVA to cases where the sample means are unequal and the distribution is not normal. The nucleotide sequences obtained in this work were submitted to the European Nucleotide Archive (www.ebi.ac.uk/ena) and are available under the accession numbers PRJEB6022 (E1) and PRJEB7591 (E2).

### Quantitative PCR

The abundance of *Enterobacteriaceae* was quantified by determining the number of 16S rRNA genes per ng of the community DNA isolated from the respective lettuce samples. The preparation of the standard fragment and the quantitative PCR was carried out following the guidelines given by Rossmann et al. [27]. The 16S rRNA gene of *Serratia plymuthica* HRO-C48 served as reference fragment for the generation of standard curves. The quantitative PCR was conducted with the ROTOR 6000 system (Corbett, Mortlake, Australia). The final reaction volume (10 μl) contained 1 μl of the 1:500 diluted template, 5 μl of 2× Kapa SYBR Fast Mastermix (Kapa Biosystems, Woburn, MA, USA) and 2.5 pmol of the primer DG47f and RW01r [35]. The cycling program was adjusted to an initial denaturation at 95°C for 10 min, followed by 40 cycles of 92°C for 30 s, 52°C for 30 s, and 72°C for 60 s. All reactions were prepared in duplicates and analyzed in two independently repeated runs. Calculated gene counts were normalized to 1 ng μl^-1^ based on the DNA concentration determined with a Nanodrop system 2000c (Thermo Scientific, Wilmington, MA, USA). Spectra which did not reach the required quality were automatically removed by the ROTOR 6000 software. Statistics were conducted using SPSS v20 PASW Statistics 18 (SPSS Inc., Chicago, IL, USA).

### Fluorescence *in situ* hybridization (FISH) and confocal laser scanning microscopy (CLSM)

FISH/CLSM was applied in two distinct approaches to both *R*. *solani* diseased (bottom rot) lettuce (rhizosphere and phyllosphere) and commercially available lettuce (*L*. *sativa* var. *capitata*) inoculated with *Escherichia coli* K12; 1.6 × 10^9^ cells ml^-1^ on 20 mm seized plant foliage discs. Root and leaf samples from both approaches were fixed with 4% paraformaldehyde (PFA) for 6 h, washed 3 times with ice-cold PBS and then stored at -20°C in 1:1 (v/v) 96% Ethanol:PBS.

FISH was performed using class specific probes following the protocol by Cardinale et al. [36]. EUB338MIX (Cy3-labeled) was used for staining overall bacterial communities. *Gammaproteobacteria* were probed with GAM42a (Cy5-labeled), *Alphaproteobacteria* with ALF968 (Cy5-labeled), and *Betaproteobacteria* with probe BET42a (ATTO488-labeled) [36,37]. All FISH probes were purchased from genXpress GmbH (Wiener Neudorf, Austria). Briefly, after pre-treatment with lysozyme, the labeled FISH probes were applied to confirm the presence and localization of the taxa detected by pyrosequencing. The FISH stained samples were further mounted with SlowFade Gold Antifadent (Molecular Probes, Eugene, OR, USA) and stored at 4°C over night until observation with a Leica TCS SPE confocal laser scanning microscope (Leica Microsystems, Mannheim, Germany) equipped with solid state and UV lasers. For each field of view, an appropriate number of optical slices were acquired with a Z-step of 0.15–0.5 μm (“confocal stacks”) and the software Imaris 7.3 (Bitplane, Zurich, Switzerland) was used for post-processing, 3D rendering and creation of isosurface-spot models.

## Results

### *Enterobacteriaceae* on lettuce

**Fig 1:**
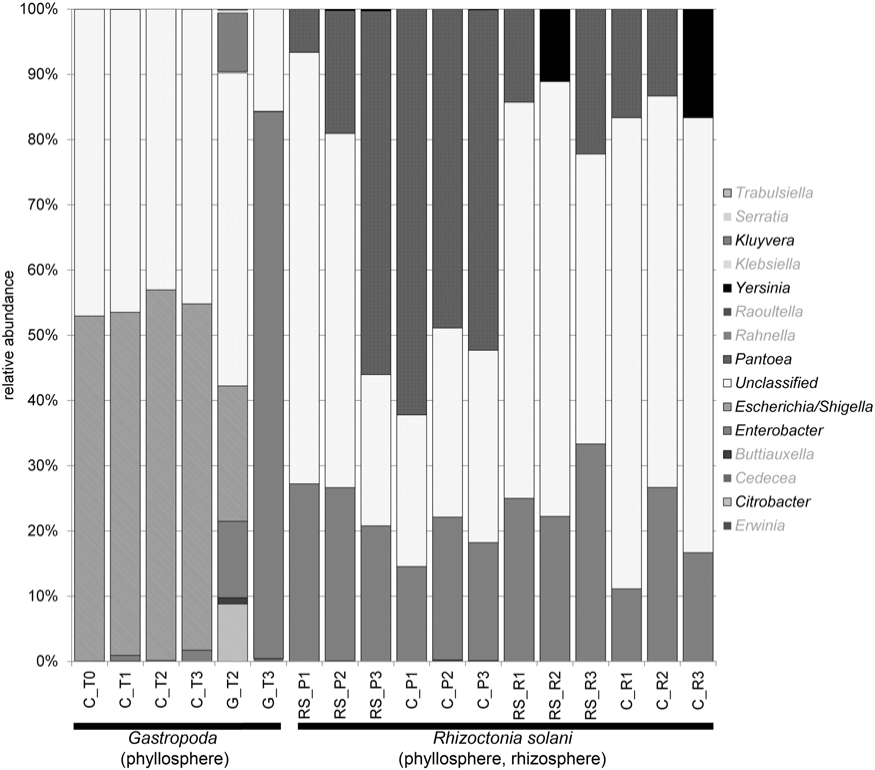
Taxonomic classification and relative abundance of *Enterobacteriaceae* associated with *L*. *sativa* var. *capitata* of phyllo- and rhizosphere samples. Genera below 1% abundance in any sample were shaded in grey (taxon legend). (Treatments; C = Control/untreated, G = *Gastropoda*, RS = *R*. *solani*; time of samplings T0–T3; P1–3 phyllosphere (replicates), R1–3 rhizosphere (replicates))

Altogether, 454 pyrosequencing of 16S rRNA gene amplicons revealed 14 distinct enterobacterial genera in the rhizosphere and phyllosphere of pot- and mesocosm-grown lettuce plants (Fig. 1). The enterobacterial microbiome contained taxa belonging to *Escherichia*/*Shigella*, *Pantoea*, *Enterobacter* and *Enterobacteriaceae* not classified at the genus level. Although these were identified as the most abundant taxa across the whole dataset, *Escherichia*/*Shigella* was only allocated to the mesocosm samples and was absent in the pot-grown samples. In contrast, we found *Pantoea* only in samples associated to the pot-grown plants from the first experiment. *Enterobacter* was the only identified taxa found in every sample and represents a core taxon. A comparison between the rhizosphere and the phyllosphere revealed that there were no significant structural differences, however the abundance of *Enterobacteriaceae* was statistically significantly higher (p<0.05) in a 3:1 ratio on phyllosphere samples determined with quantitative PCR. Interestingly, the occurrence of *Yersinia* was related to an absence of *Pantoea* in the rhizosphere.

### Impact of the fungal pathogen *Rhizoctonia solani* on the lettuce microbiome

**Fig 2:**
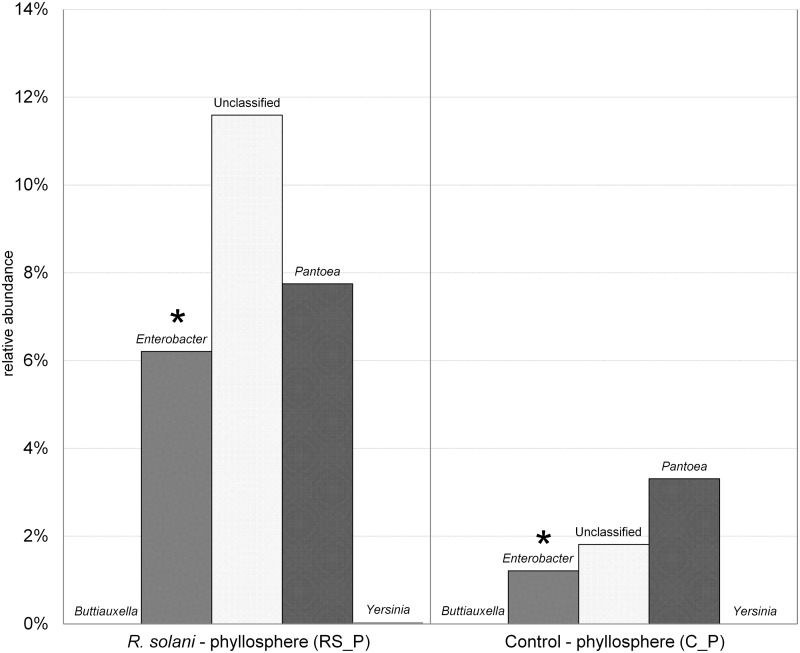
Amplicon sequencing of *Gammaproteobacteria* revealed the relative abundance of *Enterobacteriaceae* associated with *L*. *sativa* var. *capitata* phyllosphere samples of the healthy control (C_P) and plants showing symptoms of bottom rot caused by *R*. *solani* (RS_P). Asterisks: significant differences (p < 0.05) between C_P and RS_P.

*R*. *solani* inoculated lettuce plants (RS_P) with symptoms of bottom rot showed significant drifts in their associated microbial communities, and most notably a statistically significant increase from about 1% to 6% in the genus *Enterobacter* in the phyllosphere (Fig. 2). Interestingly, the structure was not significantly altered, however the genus *Enterobacter* was only found in low abundances in healthy plants (C_P). It increased significantly, likely in correlation with the spread of the pathogen on the host. A similar but less significant effect was found for *Pantoea* and other *Enterobacteriaceae* not classified at genus level (Fig. 2).

### Impact of *Gastropoda* on the lettuce microbiome

The stability of the lettuce-associated gammaproteobacterial (SSCP) and *Enterobacteriaceae* (454 pyrosequencing) community in response to biological disturbances from *Gastropoda* was followed using a mesocosm approach at four sampling times (C = Control, G = *Gastropoda*): C_T1 = start point (initial microbiota), C_T2 = adapted microbiota C/G_T3 = after five day lasting exposure to gastropods and C/G_T4 = 42 days after the exposure. Microbial fingerprints (S1 Fig.) based on separation by single strand conformation polymorphism using *Gammaproteobacteria* specific and universal primers revealed a highly stable community structure in the rhizosphere with either no, or only minor effects caused by *Gastropoda* (S1 Fig.). This was observed not only within a set of replicates at a certain time point, but also between the artificial mesocosms and between the respective samplings for both investigated taxonomic levels. However, significant structural differences and changes in the abundance levels could be found for the phyllosphere. After introduction of *Gastropoda*, new unique bands, some highly dominant, were found (G_T2). While some bands appeared transient, others were found in the same or even higher dominance 43 days after removing the *Gastropoda* from the mesocosms.

**Fig 3:**
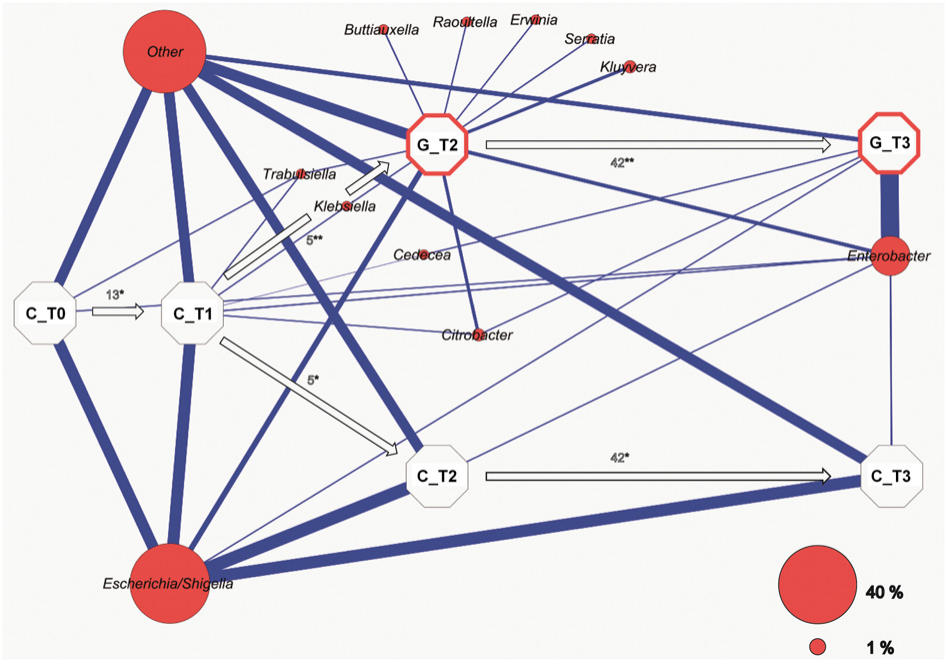
Network analysis showing the experimental process over time based on enterobacterial amplicons as resulted from 454 pyrosequencing. Each octagon represents a particular juncture (Treatment; C = Control/untreated, G = *Gastropoda*, time of sampling; T0-T3) with associated weighted average taxa (red). Edges (blue connection lines) indicate occurrence and abundance of each OTU at the connected time point. Numbers above the arrows indicate the elapsed time between the particular samplings (days). * = Steady state mesocosm ** = Mesocosm with/past snails and slugs contamination.

454 pyrosequencing of 16S rRNA gene amplicons allowed deeper insight into the indigenous microbiome at OTU based taxonomical levels. As shown in the correlation network (Fig. 3), the structure of enterics remained steady throughout the experiment at the unaffected control mesocosm. Untreated samples were dominated by *Escherichia*/*Shigella* with a relative average abundance of 52% throughout the time. The introduction of gastropods radically decreased the abundance of *Escherichia*, while *Enterobacter* increased and accumulated to a relative abundance of 84% against the total taxonomic affiliations. However, new genera (*Kluyvera*, *Erwinia*, *Serratia*, *Buttiauxella* and *Raoultella*) were also introduced, but could not be permanently established in the phyllosphere. The network once again clearly demonstrates the impact of slugs and snails primarily on the genus *Enterobacter*.

**Fig 4:**
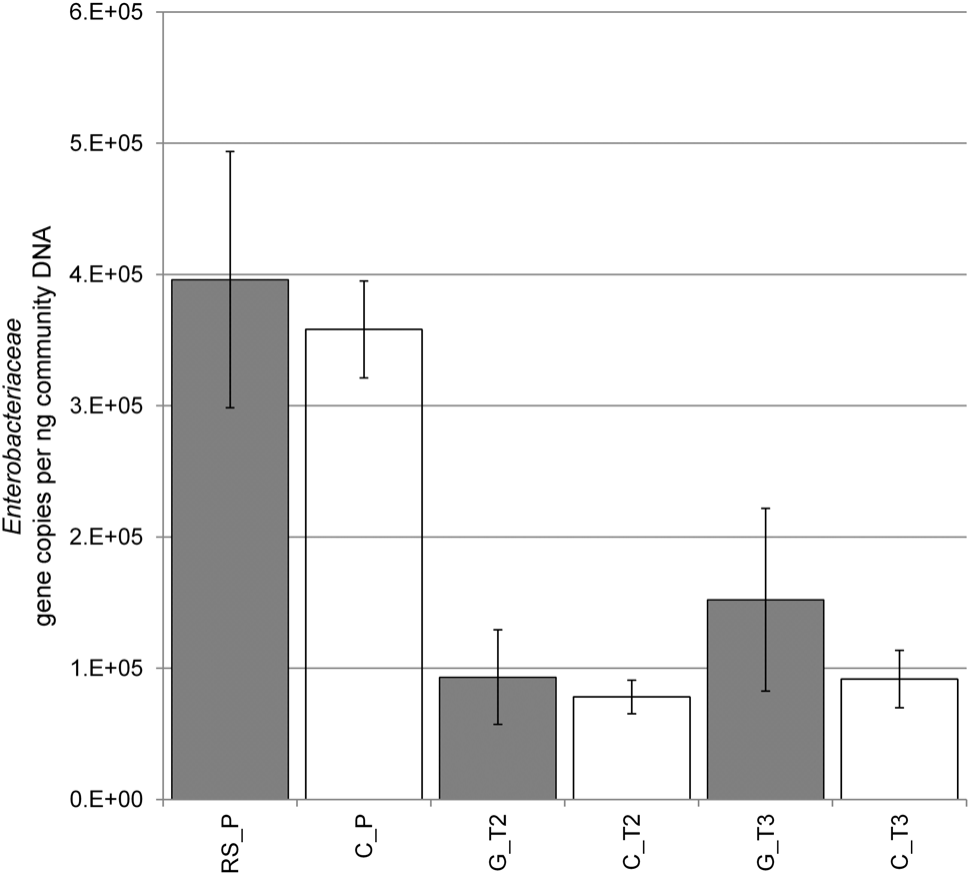
Abundance of *Enterobacteriaceae* per ng community DNA isolated from 5 g of the respective samples of the foliage. Error bars represent the confidence interval under p < 0.05. (Confidence error of the mean based on RS_P = n 18, C_P = n 19, G_T2 = n 7, C_T2 = n 7, G_T3 = n 8, C_T3 = n 8). (Treatments; C = Control/untreated, G = *Gastropoda*, RS = *R*. *solani*; time of samplings T2–T3)

Quantitative PCR supported the results of 16S rRNA amplicon sequencing of gene fragments. Significantly higher levels of enterobacteria were found when a comparison of the samples from the *R*. *solani* pot experiments (RS_P, C_P) with the samples of the *Gastropoda* mesocosms (C_T2 and T3, G_T2 and T3) was made (Fig. 4). Higher abundances of *Enterobacteriaceae* were found on lettuce samples showing bottom rot disease. Similar observations could be made analyzing the mesocosm experiments. Plants grown in the mesocosm after the biotic disturbance showed also an increase of enterobacterial taxa when compared to the control. The enterobacterial abundance of the root was 1.50 × 10^5^ gene copies ng^-1^ compared to 3.77 × 10^5^ gene copies ng^-1^ community DNA retrieved of the foliage.

### Lettuce leaves as niches for bacteria visualized by microscopy

**Fig 5:**
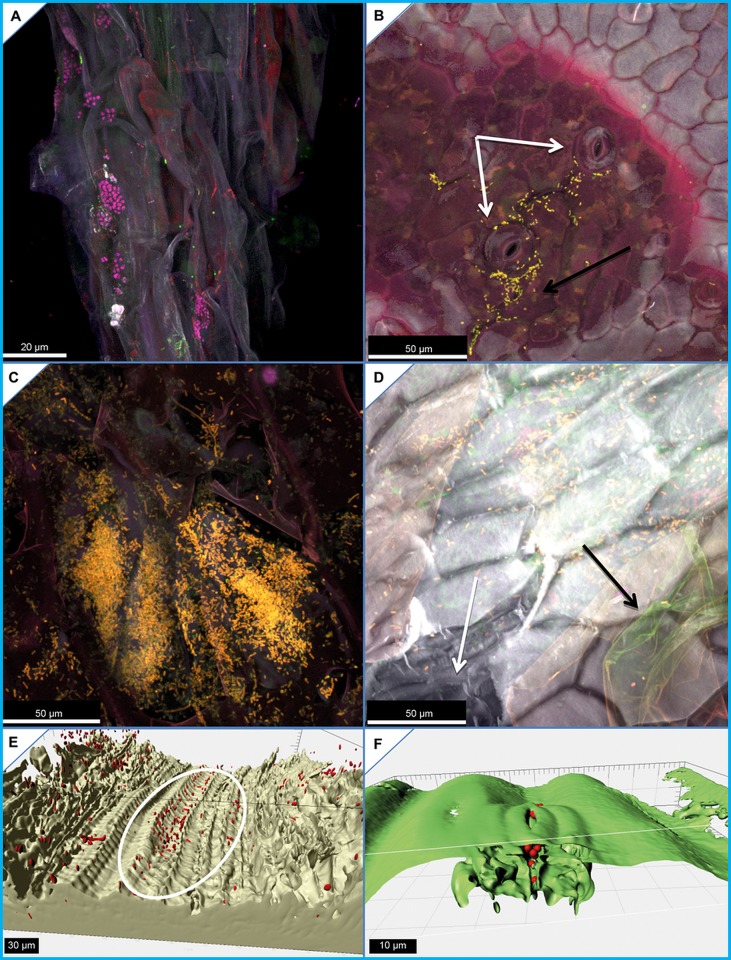
Fluorescence *in situ* hybridization coupled with confocal laser scanning microscopy images. A) *Gammaproteobacteria* (yellow) and *Betaproteobacteria* (pink) form large colonies on the lateral roots of young lettuce plantlets, where they do not share their space, moreover it seems they exclude each other. B) We revealed increased gammaproteobacterial (yellow) colonization close to stomata which could be also detected on *R*. *solani* infected lettuce. C) Punctiform extensive colonization behavior of *Escherichia coli* K12 (yellow) on inoculated lettuce patches within the inner compartments of the leafy green. D) Physical damage to the leaves affects the epicuticular wax-layer (black arrow) and the cuticle cells (white arrow), and supports endophytic colonization of enterobacteria (yellow). E) Transport of bacteria (red) within a vascular bundle (white marker) and F) trough stoma.

All samples from the first experiment were microscopically analyzed to validate our sequencing results and to reveal colonization patterns on lettuce. In all experiments, the lettuce roots were found densely colonized by bacterial colonies which were taxonomically differentiated using specific FISH probes (Fig. 5). The root surface was completely colonized by bacteria. *Gamma*- and *Betaproteobacteria* formed dominant colonies on the lateral roots of young lettuce plantlets, and after disturbances the increase of *Enterobactericeae* could be confirmed microscopically. However, it was difficult to visualize the phyllosphere bacteria on the leaf surface as only a few colonies could be detected, usually concentrated in the surrounding of stomata (Fig. 5B). This observation was directly in contrast to the diversity shown at molecular level. To find the reason for these contradicting results, we analyzed leaves, which were treated with bacterial cell suspensions of *E*. *coli* K12. Surprisingly, we observed large colonies and substantial punctiform colonization in endophytic compartments (Fig. 5C). In the inoculation experiment, we could not identify epiphytic bacterial colonization; instead micrographs showed that bacteria colonize the inner leaf compartments through i) bruises or physical damage (Fig. 5D), ii) the endovascular systems (Fig. 5E), and iii) through stomata (Fig. 5F).

## Discussion

Our study identified the structure of *Enterobacteriaceae* on and in lettuce leaves and roots and described an impact of biotic stress on their diversity and abundance. The latter result confirmed our hypotheses that biotic disturbances induce shifts in the *Enterobacteriaceae* plant-associated community, and that each pathogen cause significant changes within the bacterial community.

All investigated lettuce plants harbor phyllo- as well as rhizosphere bacteria, which belong to the *Enterobacteriaceae* family. The spectrum was similar to the data published for the lettuce phyllosphere [2,4,5]. Quantitative PCR revealed a higher abundance in the phyllosphere than in the rhizosphere in general as well as an enhancement in response to biotic disturbances. An abundance scatter plot presenting the distribution of the samples revealed that there is not only an increase of abundance of enterobacteria, but also an elevated inhomogeneity across the samples in a disturbed sample pool. Interestingly, samples derived from the pot-grown, healthy lettuce, but also plants from the control mescosm showed much smaller scattering within the comparable samples.

We were able to identify similar *Enterobacteriaceae* species in the rhizosphere and phyllosphere of same lettuce plants and found structural differences between plants grown in mesocosm and in the pot experiments. In general, due to different abiotic conditions in the upper and below-ground parts of plants, these microhabitats are colonized by specific microbial communities and shared only a few ubiquitous species [10,38]. However, the phyllosphere of lettuce is also influenced by the soil microbiome [6].

First, it was difficult to visualize phyllosphere bacterial communities in our microscopic study. Only a few bacterial colonies could be seen on the leaf surface, while the root surface was densely colonized. We assumed that hydrophobicity and/or antimicrobial substances of epicuticular waxes prevent excessive bacterial growth. The leaf surface of a plant, especially its chemical components, constitutes the first line of resistance against herbivores and other pests and pathogens, and also acts as a shield against UV rays. The impact of plant cuticular wax composition was already shown to affect the community composition of phyllosphere bacteria in *Arabidopsis* mutants [39]. After a microscopic survey investigating several respective samples, we found that the bacteria are not equally distributed; moreover they tend to colonize micro-niches on the leaves. To evaluate our results, we inoculated lettuce leaves with a bacterial suspension of *E*. *coli* K12 that confirmed the native colonization patterns and showed that the inner tissues below the waxes were densely colonized. Our finding is congruent with the observation of Franz et al. [40], who observed the presence of the pathogens in lettuce after thorough surface sterilization. They demonstrated the presence of human pathogens on lettuce, which were unlikely to be removed by the actions of consumer washing and therefore pose a serious threat when occurring in agriculture or home gardening situations.

The biotic stresses studied in this study induced significant shifts in the bacterial community and increased species richness, although they caused only slight (*Rhizoctonia*) or no symptoms (*Gastropoda*) on the lettuce plants. According to the intermediate disturbance hypothesis described in macroecology [41], diversity should be maximized at intermediate levels of disturbance because both competitive K-selected and opportunistic r-selected species can coexist. This hypothesis has been a matter of controversy in macroecology [42], and cannot be applied in general to microbial communities. It is notable that enterobacteria, which were found in higher abundances after disturbance, belong primarily to r-selected species and are well-known for their opportunistic and fast-growing character. If we consider the impact of fungi and gastropods as intermediate disturbance, then we would see support for an intermediate disturbance hypothesis by the general increase in species richness.

The shift within the *Enterobacteriaceae* family can be of importance for human health due to the connection of plant associated biodiversity and the human immune system. Recently, Hanski et al. [43] showed a correlation between bacterial diversity and atopy, most likely from significant interactions with *Gammaproteobacteria*. Endotoxin derived from Gram-negative bacteria, such as *Enterobacteriaceae*, is known to have allergy-protective and immunomodulatory potential [44]. Therefore, the natural occurrence and enhancement of enterics by biotic stress may have an impact on our immune system and health, which requires further investigation. Moreover, we have shown that enterics tend to invade the plant tissue, although the intact epicuticular wax layer seems to protect the leafy green portion from microbial colonization. We also argue that the high abundance and frequent appearance of opportunistic pathogens is contradictory to the relatively low number and frequency of serious outbreaks. With this knowledge, we suggest the enrichment of enterobacteria might have commensal or even immunomodulatory effects and could act as a “natural vaccine”. In particular we hypothesize that the consumption of fresh food such as lettuce may increase general human well-being, and possibly enrich the commensally gastrointestinal microbiota to recurrently stimulate the immune system. The potential priming effect could be an important additional attribute of lettuce as healthy raw food, and in general with regards to nutritional regimens based on consumption of raw plant parts.

## Supporting Information

S1 Fig Band based analysis using dice algorithm of microbial fingerprints showing the structure of all bacteria and *Gammaproteobacteria* of *L*. *sativa* from the mesocosm experiment. (TIF)
