# Supplementary figures and images for: Biotic Stress Shifted Structure and Abundance of Enterobacteriaceae in the Lettuce Microbiome
Source: PLoS One. 2015 Feb 25;10(2):e0118068. doi: 10.1371/journal.pone.0118068 (PMC4340628; doi:10.1371/journal.pone.0118068)

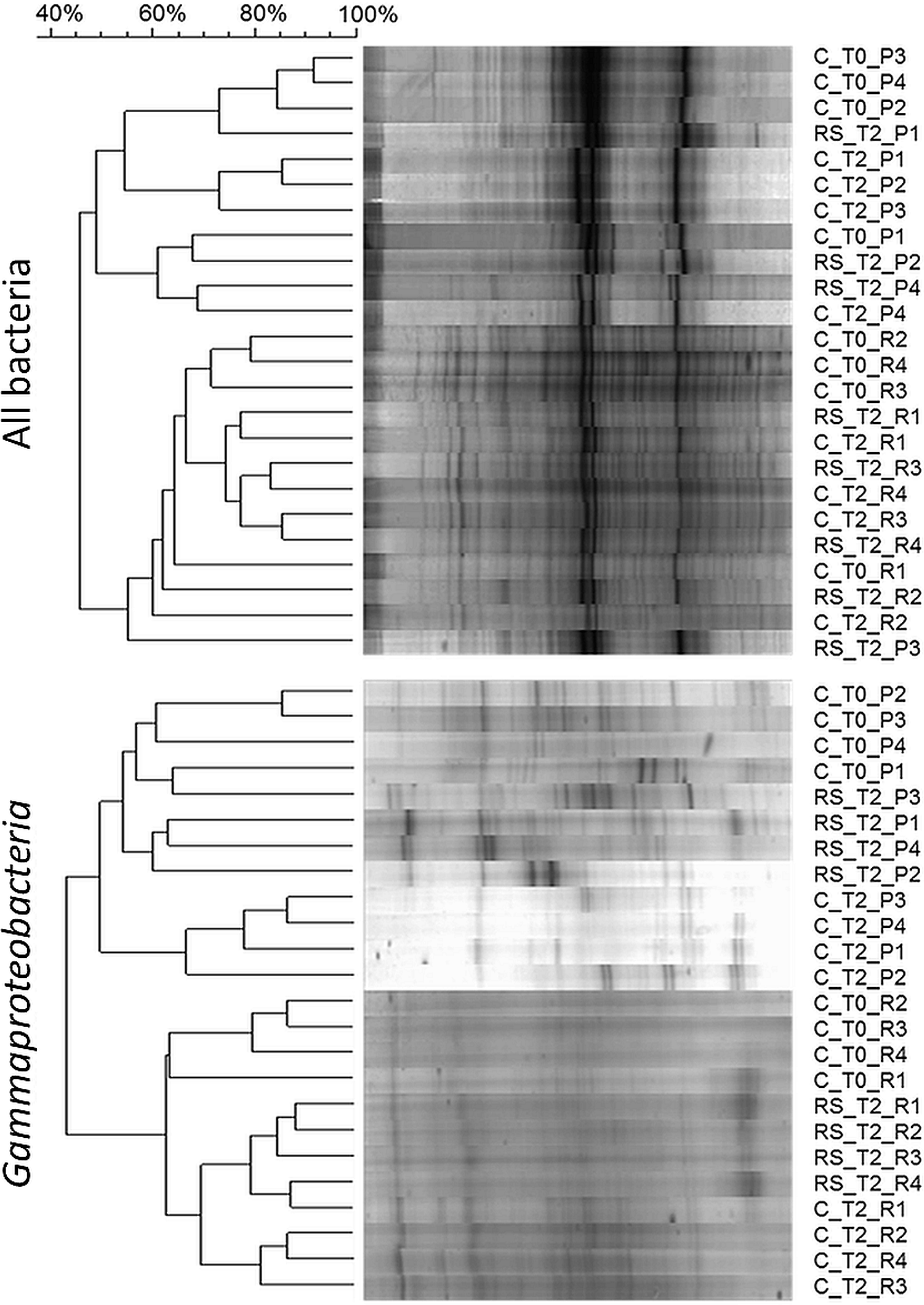

Supplement: S1 Fig — (TIF) [file pone.0118068.s001.tif]
